# Mechanical birth-related trauma to the neonate: An imaging perspective

**DOI:** 10.1007/s13244-017-0586-x

**Published:** 2018-01-22

**Authors:** Apeksha Chaturvedi, Abhishek Chaturvedi, A. Luana Stanescu, Johan G. Blickman, Steven P. Meyers

**Affiliations:** 1 Department of Imaging Sciences, University of Rochester Medical Center, 601, Elmwood Avenue, Box 648, Rochester, NY 14642 USA; 2 Department of Radiology, Seattle Children’s Hospital, Seattle, WA USA

**Keywords:** Neonate, Mechanical trauma, Macrosomia, Instrumental delivery, Cephalopelvic disproportion

## Abstract

Mechanical birth-related injuries to the neonate are declining in incidence with advances in prenatal diagnosis and care. These injuries, however, continue to represent an important source of morbidity and mortality in the affected patient population. In the United States, these injuries are estimated to occur among 2.6% of births. Although more usual in context of existing feto-maternal risk factors, their occurrence can be unpredictable. While often superficial and temporary, functional and cosmetic sequelae, disability or even death can result as a consequence of birth-related injuries. The Agency for Healthcare research and quality (AHRQ) in the USA has developed, through expert consensus, patient safety indicators which include seven types of birth-related injuries including subdural and intracerebral hemorrhage, epicranial subaponeurotic hemorrhage, skeletal injuries, injuries to spine and spinal cord, peripheral and cranial nerve injuries and other types of specified and non-specified birth trauma. Understandably, birth-related injuries are a source of great concern for the parents and clinician. Many of these injuries have imaging manifestations. This article seeks to familiarize the reader with the clinical spectrum, significance and multimodality imaging appearances of neonatal multi-organ birth-related trauma and its sequelae, where applicable.

*Teaching points*

• *Mechanical trauma related to birth usually occurs with pre-existing feto-maternal risk factors*.

• *Several organ systems can be affected; neurologic, musculoskeletal or visceral injuries can occur*.

• *Injuries can be mild and transient or disabling, even life-threatening*.

• *Imaging plays an important role in injury identification and triage of affected neonates*.

**Electronic supplementary material:**

The online version of this article (10.1007/s13244-017-0586-x) contains supplementary material, which is available to authorized users.

## Introduction

The process of birth, whether spontaneous or assisted, is inherently traumatic for the newborn. Birth-related injuries encompass both mechanical and hypoxic-ischemic events. This review focuses mostly on mechanical trauma sustained by the neonate owing to the forces of labor and delivery. For conciseness of this review, birth-related hypoxic-ischemic injuries to the neonate will not be separately addressed.

Trauma related to birth may affect several organ systems of the neonate (ESM_1). The exact incidence of mechanical trauma of birth may be somewhat underestimated. Incidence is 0.82%, prevalence has been estimated at 9.5 per 1000 live births [1]. Less than 2% of neonatal deaths result from birth trauma [2].

Birth-related trauma can occur without identifiable risk factors; however, it is more common in context of predisposing feto-maternal risk factors. Risk factors can be fetal (macrosomia-birth weight > 4500 g, malpresentation or shoulder dystocia (defined as passage of more than 60 s between the delivery of the head and body [3], resulting in requirement of additional obstetric maneuvers for delivery of fetal shoulders [4])); maternal (diabetes, primiparity, small pelvis); or obstetric (epidural analgesia, induced or instrumental delivery).

Over the following paragraphs, we discuss the clinical context and imaging findings of birth -related injuries categorized by different portions of the neonate’s anatomy.

## Injuries to the head and face

### Extracranial

#### Scalp

**Fig. 1:**
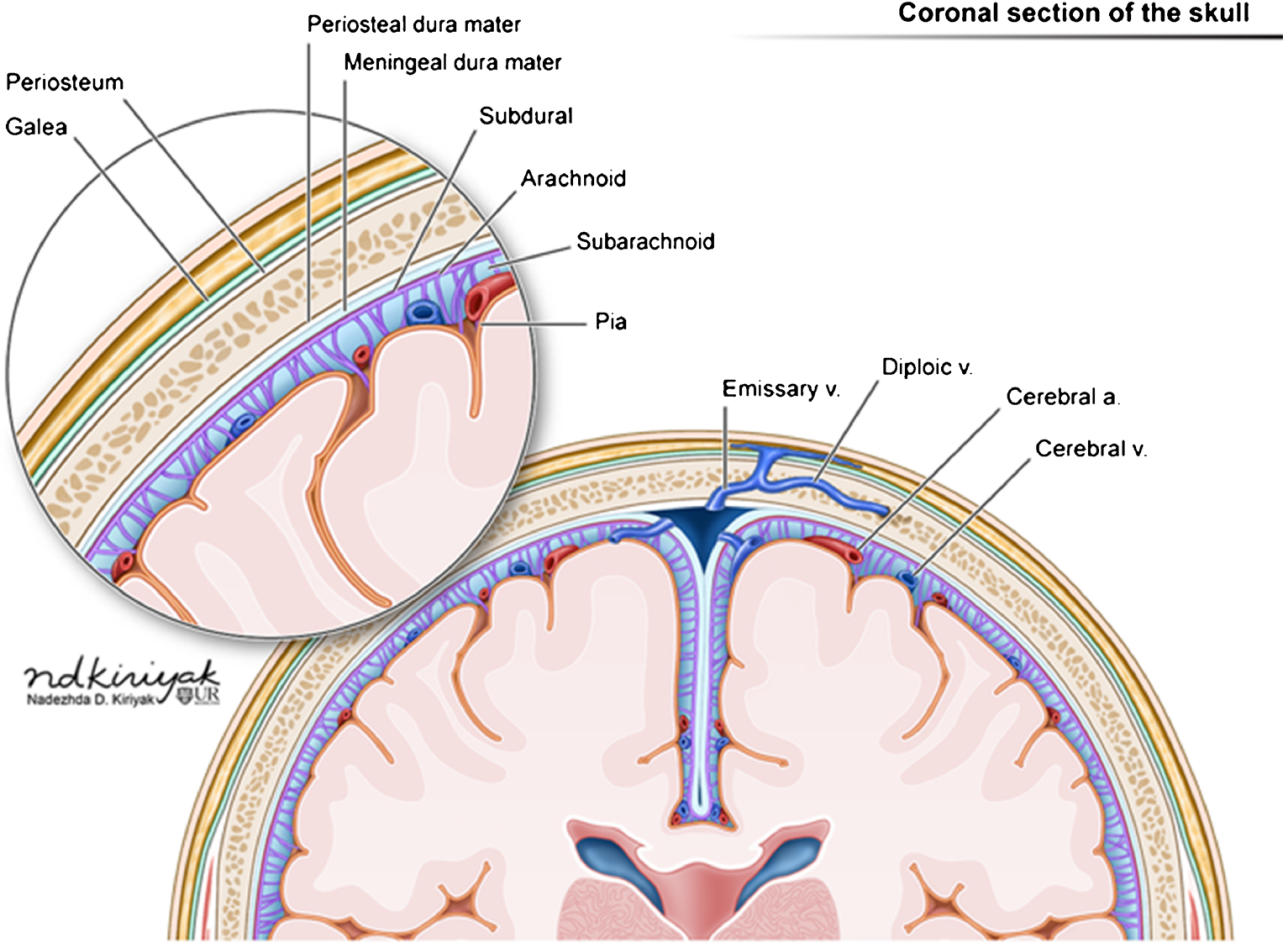
Illustration demonstrating the layers of scalp, skull, meninges and brain on a coronal section (a)

The different layers of scalp are skin, subcutaneous connective tissue, galea aponeurotica, loose areolar connective tissue and periosteum. Normal anatomy of the scalp is depicted with illustrations (Fig. 1).

**Fig. 2:**
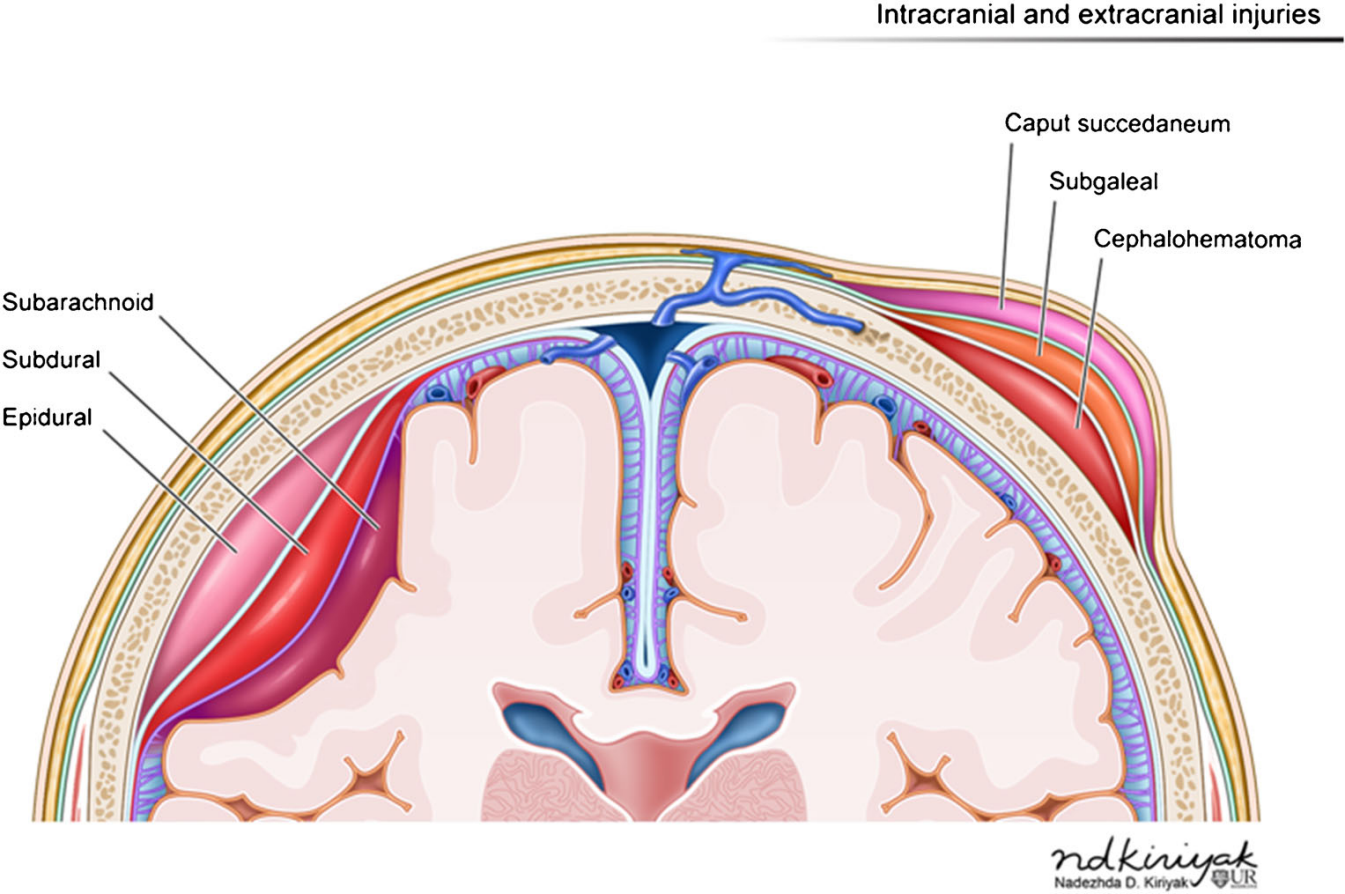
Illustration depicting hemorrhages by location within the different layers of the meninges (left of image) and scalp (right of image)

**Fig. 3:**
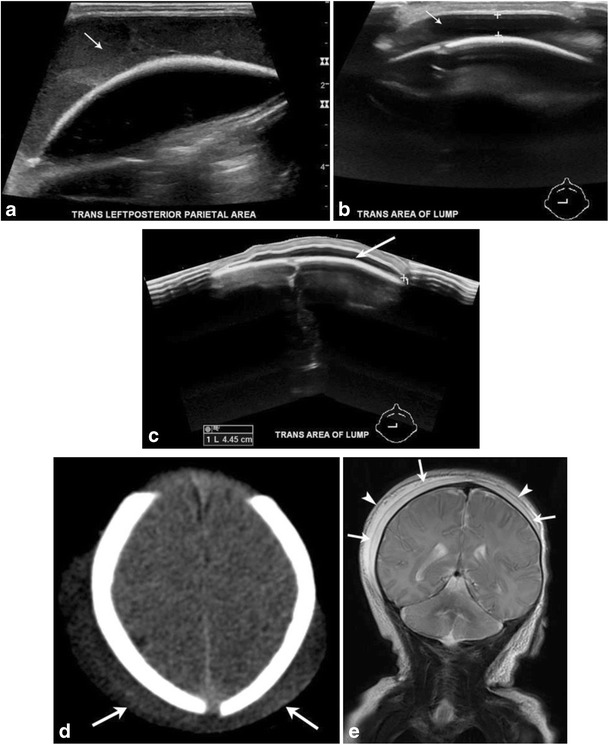
Caput succedaneum and subgaleal hemorrhage Grayscale ultrasound images (a-c) of the scalp in a newborn male demonstrate a fluid collection that crosses midline, is deep to the subcutaneous fat/galeal aponeurosis and superficial to the periosteum/calvarium (noted as thick echogenic interface) consistent with a subgaleal hemorrhage. Axial CT (d) image in a 1-day-old male with history of traumatic delivery demonstrate scalp soft tissue overlying bilateral parietal regions and crossing the sagittal suture (arrows). Follow-up coronal MR image (e) demonstrates a deep subaponeurotic scalp fluid collection crossing the sagittal suture and extending anteriorly into the right temporal region, consistent with subgaleal hematoma (arrows). A more superficial overlying fluid collection with a similar distribution also noted (arrowheads). This collection is within the subcutaneous fibrofatty tissues superficial to galea aponeurosis and is consistent with caput succedaneum

**Fig. 4:**
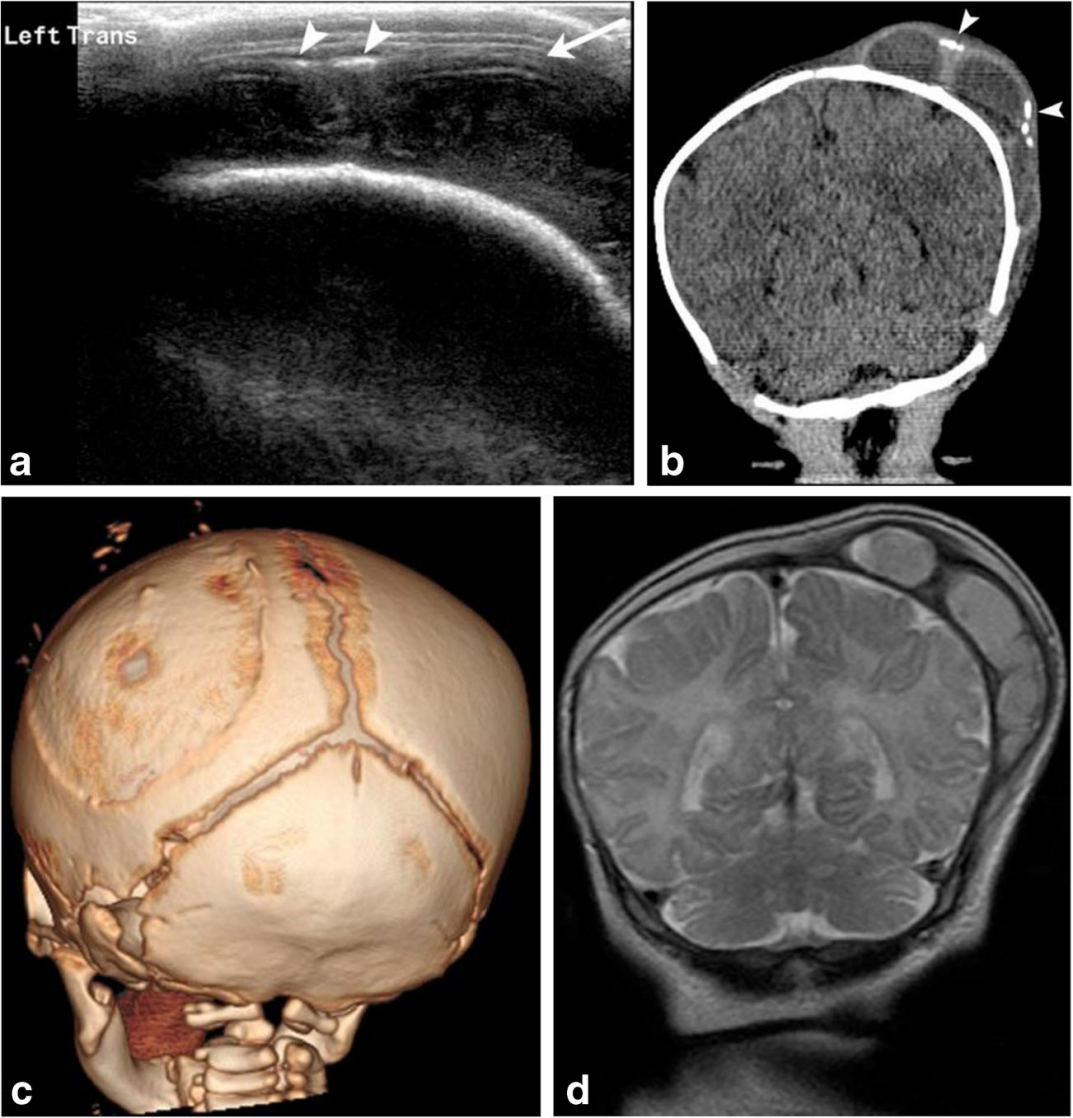
Cephalhematoma 1-month-old male with history of traumatic delivery presenting with right parietooccipital soft tissue swelling. Transverse grayscale ultrasound (a) image of the left parietooccipital scalp shows a complex fluid collection (arrow), with punctate linear echogenic foci along the superficial aspect (arrowheads), suggestive of calcifications. Relationship with the adjacent left lambdoid suture was difficult to evaluate by ultrasound. B. Coronal non-contrast head CT (b) image demonstrates a lobulated fluid collection with thick septations and peripheral calcifications (arrowheads) that does not cross the adjacent sagittal or the lambdoid suture, suggestive of cephalhematoma. 3-D volume (c) rendered images in bone algorithm shows cortical irregularity along the left parietal bone at the site of cephalhematoma as well as peripheral calcifications along the superficial aspect of the cephalhematoma. Coronal T2 image (d) from an MR exam obtained one week later in the setting of patient’s seizures re-demonstrates the large subperiosteal complex fluid collection with thick septations and isointense fluid signal consistent with evolving blood products in the known left parietal cephalhematoma

Hemorrhages may occur within different layers of the scalp and meninges (Fig. 2). The main categories of scalp hemorrhages include caput succedaneum (Fig. 3d-e), subgaleal hemorrhage (Fig. 3a-e) and cephalhematoma (Fig. 4a-d). These traumatic extracranial lesions each have their unique clinical presentation and course (ESM_2). The diagnosis is usually clinical; imaging plays a supplemental role. Majority of these hemorrhages spontaneously resolve with little clinical consequence. However, extensive blood loss into the subgaleal space can occasionally occur, which necessitates blood transfusion and surgical evacuation of the hematoma [5].

#### Skull

**Fig. 5:**
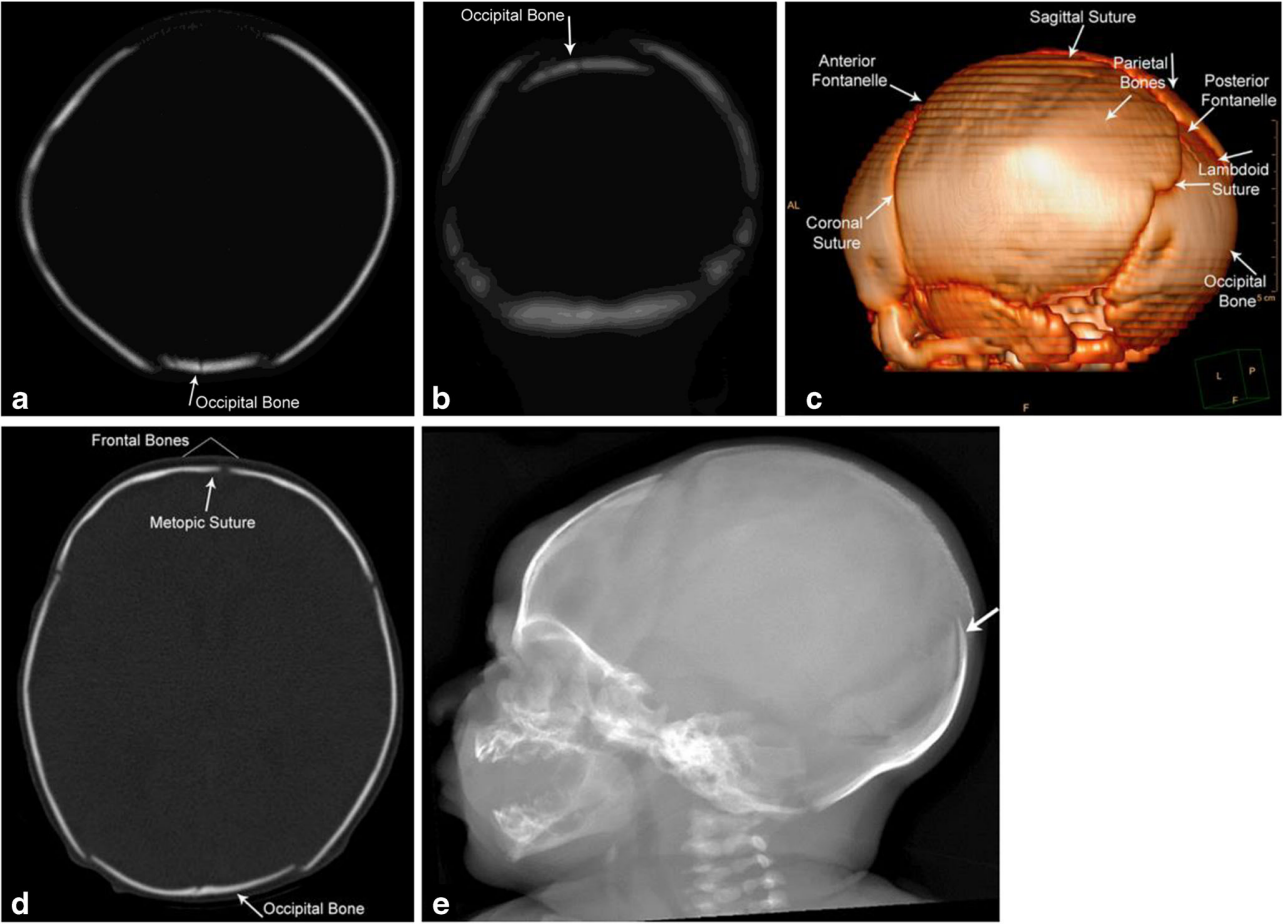
Molding of the skull post vaginal delivery Immediate post-delivery appearances of the skull on head CT. The occipital bone is slightly depressed with associated sutural overlap as seen on the axial and coronal CT images and the 3-D reconstructions (a-d). Lateral skull radiograph (e) demonstrates overlap of the occipital bone (white arrow)

The neonatal skull is composed of multiple partially ossified bony and cartilaginous components separated by sutures, synchondroses and fontanels [6]. During its passage through the birth canal, the fetal head undergoes “molding” according to maternal pelvic dimensions (Fig. 5a-e). When the head is the presenting body part, the frontal and occipital bones are compressed, leading to parietal bones being displaced outward, resulting in a step-off between the coronal and lambdoid sutures and slight widening of the squamous suture [1]. With the less common breech, brow or face presentations, however, the parietal bones are pressed inward. In either instance, if the deformation occurs rapidly or severely, the falx, tentorium or bridging veins may tear, leading to intracranial hemorrhages [1]. Similarly, the process of molding may lead to distortion of synchondroses at the skull base, with long-term consequences such as basilar impression, atlanto-occipital assimilation or nuchal impression [1].

**Fig. 6:**
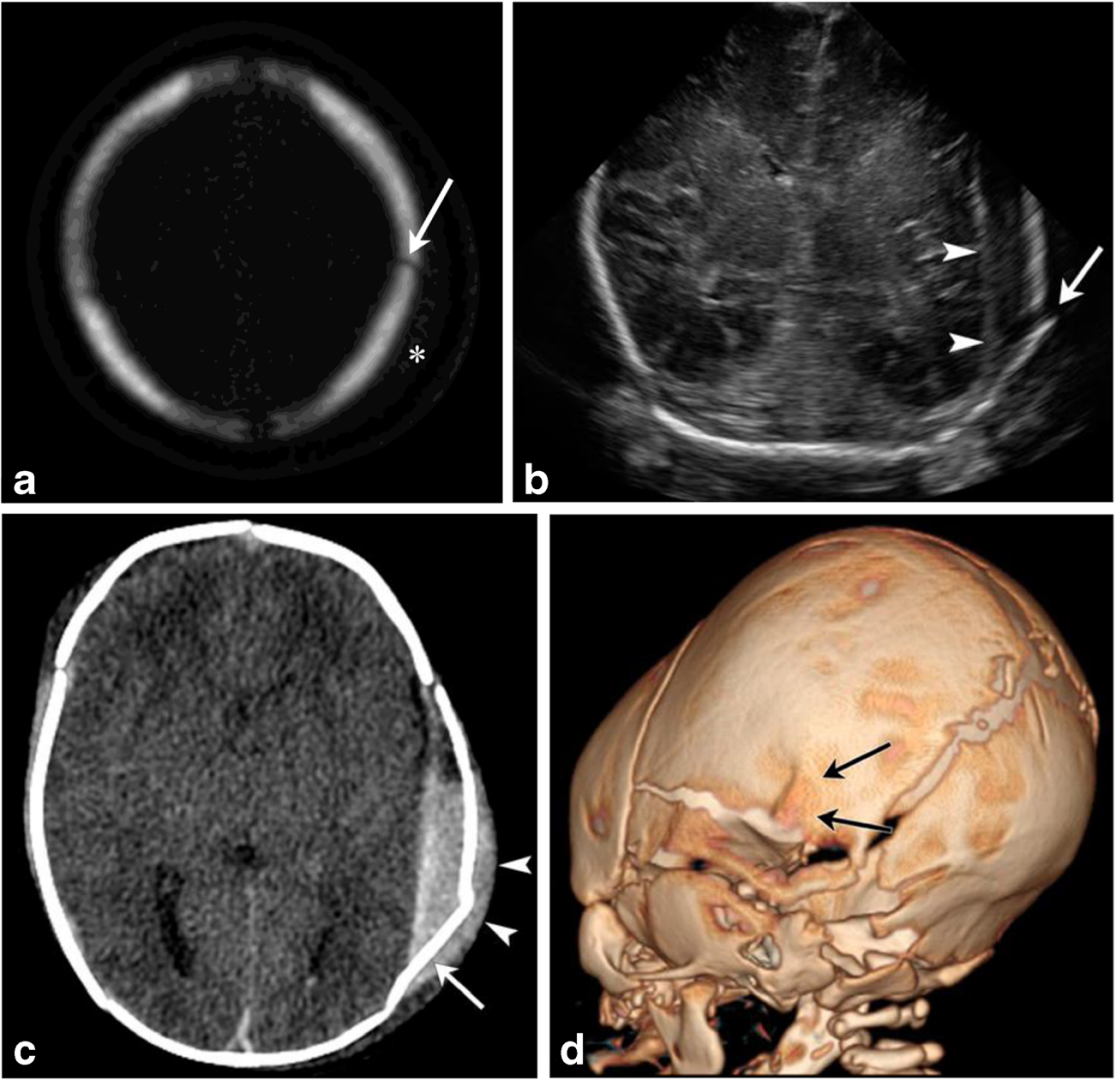
Skull fractures in two neonates Axial bony algorithm reconstruction from a head CT (a) in a 2-day-old demonstrates a non-displaced linear left parietal skull fracture (arrow) with overlying soft tissue swelling (marked by asterisk on a). Ultrasound and CT images on another 1-day-old male (b, c) with a history of traumatic delivery characterized by multiple attempts of vacuum extraction. Coronal gray scale ultrasound image (b) demonstrates a displaced left parietal fracture (arrow) with underlying extra-axial fluid collection (arrowheads). Axial non-contrast head CT image (c) shows a complex left parietal bone fracture with an angulated anterior component and an adjacent depressed “ping-pong” fracture component posteriorly (arrow). There is an associated overlying hyperdense fluid collection consistent with cephalhematoma (arrowhead). There is also an underlying large epidural hemorrhage with fluid/fluid levels. 3-D volume rendered image (d) re-demonstrates the complex left parietal bone fracture (black arrows)

Skull fractures rarely occur with traumatic birth. The commonly described fracture patterns are linear (Fig. 6a), depressed (Fig. 6b,c) and occipital osteodiastasis (ESM_3). Neonatal depressed skull fracture implies inward buckling of the very soft neonatal skull and not bony discontinuity. Occipital osteodiastasis, implying separation of squamous and lateral portions of the developing occipital bone, occurs secondary to pubic symphyseal pressure against the suboccipital region, with breech infants especially vulnerable [1]. All these fractures can be associated with intra- and extra-cranial hematomas (Fig. 6b-d). CT with multiplanar and 3D reconstructions is an excellent tool for diagnosis of these fractures and associated hematomas, with MR considered if findings on CT do not offer sufficient explanation for patient’s symptoms [6].

**Fig. 7:**
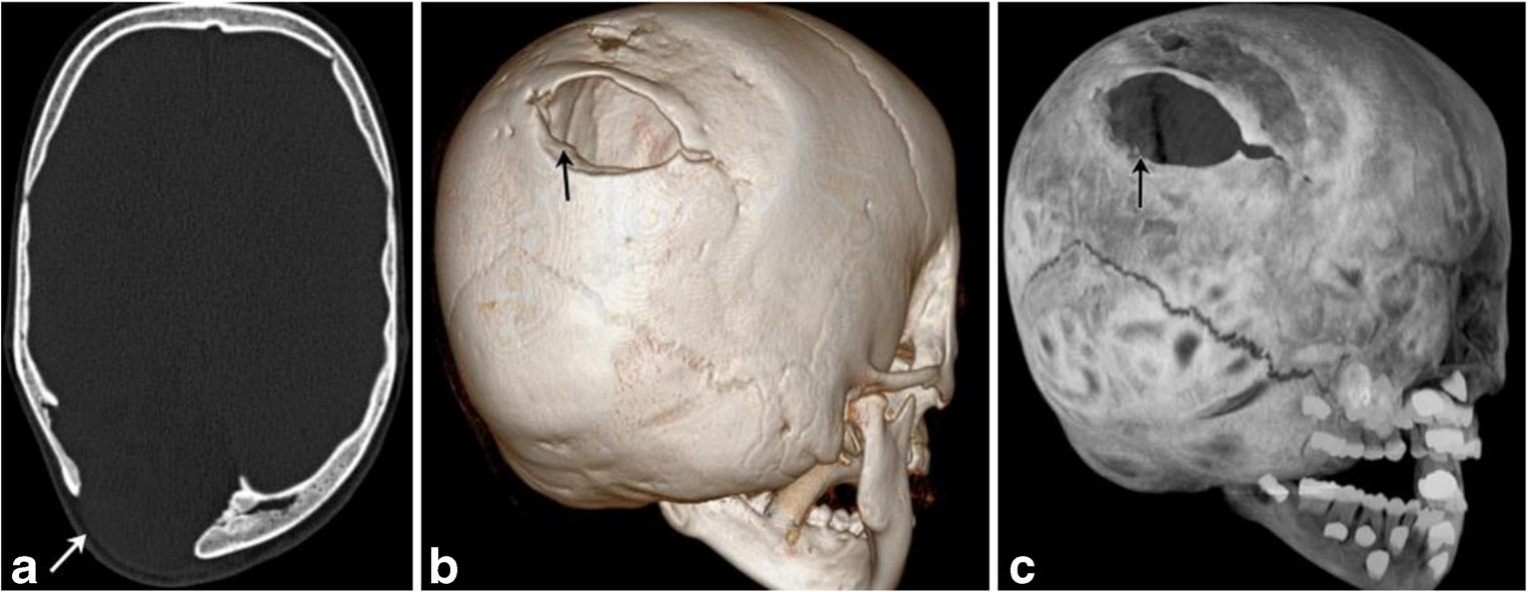
Leptomeningeal cyst Axial skull CT (a) in a now 6-year-old with a history of traumatic birth and subsequent cerebral palsy. He had a right posterior parietal calvarial fracture at birth, which did not heal, but enlarged secondary to entrapment of leptomeninges at the fracture site—an entity called growing fracture or leptomeningeal cyst [volume rendered (b), and MIP 3D reconstruction (c)]. Bony margins at the fracture site are scalloped and smooth

Leptomeningeal cysts or growing fractures are a unique entity seen among children (Fig. 7a-c), where there is progressive enlargement of the fracture secondary to CSF pulsations from injured leptomeninges entrapped in the skull defect [6]. Bony edges of the fracture are smooth/scalloped [6]. Clinically, a scalp mass is appreciated. High resolution head ultrasound can be performed as the initial imaging, followed by CT or MRI [6].

### Intracranial

**Fig. 8:**
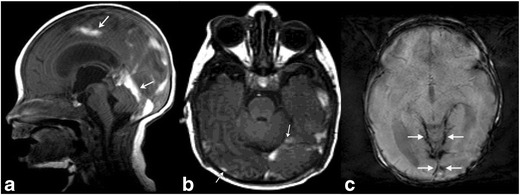
Subdural Hematoma Brain MRI in a 6-day-old term neonate with history of vacuum-assisted delivery. Sagittal T1 (a) MR image demonstrates subdural hematomas tracking along bilateral occipital lobes and along the tentorium. Another 8-day-old term neonate with history of prolonged rupture of membranes shows subdural hemorrhage (b) layering along the occipital-parietal convexities and along the tentorium (arrows). Corresponding axial gradient-recalled echo (susceptibility-weighted) image (c) at a slightly more cephalad level reveals hemosiderin staining along the tentorium and posterior convexities of the brain (arrows)

**Fig. 9:**
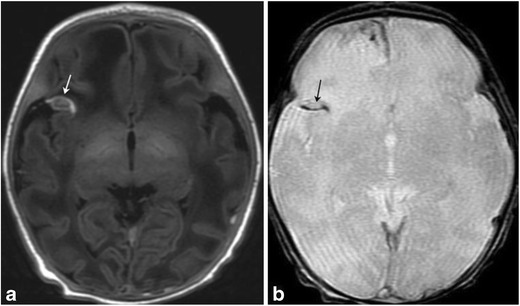
Subarachnoid hematoma Axial T1/GRE MRI in a 9-day-old term neonate with history of difficult, vacuum-assisted delivery. Right frontal blood is noted in a gyriform distribution, suggesting subarachnoid blood. Subdural blood was also noted tracking along the falx

**Fig. 10:**
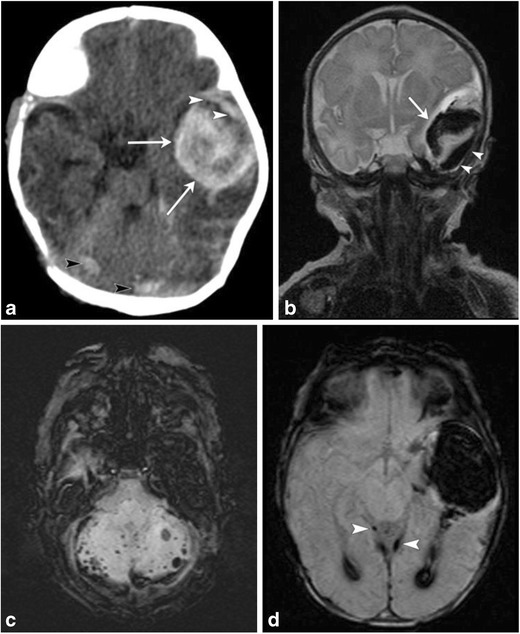
Extraaxial, intraventricular and parenchymal hemorrhages in 3-day-old female post-complicated vaginal delivery presenting with seizures Axial non-contrast head CT (a) and coronal T2 MR image (b) show a large left temporal parenchymal hemorrhage (arrows), with an overlying small subdural hemorrhage (white arrowheads). Small foci of subarachnoid hemorrhage noted along the posterior fossa (white arrowheads on a), with susceptibility artifact on axial susceptibility-weighted image (SWI) (c). Axial SWI MR image (d) through the left temporal hemorrhage also demonstrates blood products within occipital horns of bilateral lateral ventricles and trace subdural hemorrhage along the tentorium (white arrowheads). Follow-up MR at 3 months (not shown) did not demonstrate an underlying left temporal parenchymal mass or vascular malformation

Traumatic birth-related intracranial hemorrhages can occur both into the extra-axial spaces [epidural (Fig. 6b,c), subdural (Fig. 8a-c) and subarachnoid (Fig. 9a,b)] and within the cerebral or cerebellar parenchyma (Fig. 10a-d) (ESM_4). Besides large parenchymal bleeds, small cortical contusions and shear or axonal injuries may also be seen with birth-related trauma [7].

**Fig. 11:**
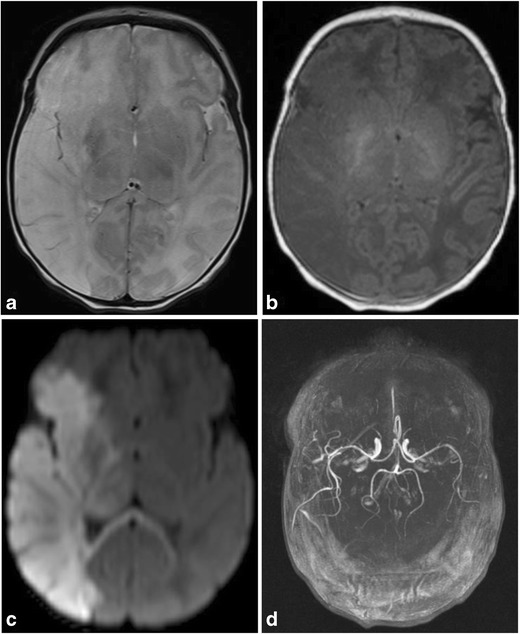
Middle cerebral artery infarct in a 4-day-old male with history of prolonged delivery with nuchal cord presenting with seizures Axial T2 (a), T1(b) and diffusion MR (c) images demonstrate an extensive region of hypointense T1 and hyperintense T2 signal involving right cerebral hemisphere in the distribution of the middle cerebral artery, with effacement of the sulci and right lateral ventricle, with corresponding restricted diffusion, consistent with right MCA distribution subacute ischemic infarct. Restricted diffusion also extends into the right thalamus, basal ganglia, cerebral peduncle and across the splenium of the corpus callosum. Axial MIP image (d) from a 2 D time-of-flight MR angiogram of the head without contrast shows normal intracranial arteries with no evidence of decreased or absent flow in the right MCA

Rarely, arterial stroke can also result from either direct trauma to a large vascular structure, compression injury from a large extraaxial bleed or stretching of arteries from forces of labor and delivery (Fig. 11a-d) [6].

Based on a study of asymptomatic neonates following full-term spontaneous vaginal birth [8], the prevalence of intracranial hemorrhage was estimated to be 26%. These hemorrhages were not associated with signs of overt trauma. The majority of these hemorrhages were found to be subdural and infratentorial. These were found to be without clinical consequence [8, 9]. Also, these hemorrhages were all of the same age [8]. The pterion is a large, relatively unprotected sutural confluence, which makes this site vulnerable for injury [10]. MR is superior to CT for evaluation of extracerebral and posterior fossa hemorrhages [8]. Susceptibility weighted imaging is especially useful for delineation of both intra- and extraaxial hemorrhages [11]. Supratentorial intracerebral hemorrhages are well seen and can be dated with both CT and MR, although ultrasound can be useful for initial bedside evaluation [6].

### Face

**Retinal hemorrhages** are seen among one-quarter of otherwise normal deliveries, but instrumental delivery and cord around the neck have been identified as risk factors [12]. Spontaneous vaginal delivery, prolonged second stage of labor and neonatal intracranial hemorrhage can exacerbate these hemorrhages [12, 13]. In one prospective study [14], all detected birth-related retinal hemorrhages resolved by 1 month of age [14]. Coexistence of these hemorrhages with skull fractures/intracranial hemorrhages secondary to mechanical birth trauma can lead to confusion with nonaccidental trauma.

Passage through the birth canal may lead to facial trauma including mostly abrasions of the face, although traumatic luxation of the nose [15] and neonatal nasal septal deviation [16] have been reported as a consequence of birth-related trauma.

## Injuries to the spinal cord and neck

**Fig. 12:**
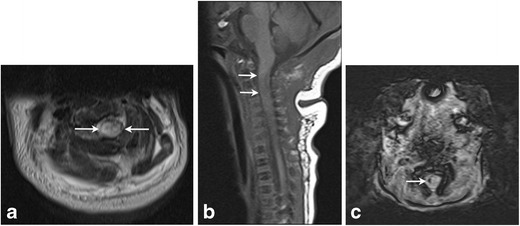
Spinal cord injury in 4-day-old female with a history of shoulder dystocia presenting with right sided upper and lower extremity neurologic deficits Axial T2 (a) and sagittal T1 (b) images of the cervical spine demonstrate a focal area of T1 hyperintensity and T2 hypointensity (arrows) in the high cervical cord at the level of C2–3 consistent with acute injury. Focal hemorrhage in the right cervical cord with susceptibility artifact seen on C (axial SWI). Finding was thought consistent with spinal cord injury secondary to stretching/traction

Spinal cord injuries are rare conditions, which may occur in context of difficult delivery characterized by excess traction, rotation and hyperextension (Fig. 12a-c) [17]. Breech presentation complicated by entrapped fetal head has been found to be responsible for many reported cases [18]. Vertebral fractures or spinal dislocations can be associated [18]. A lateral radiograph of the spine should be obtained to demonstrate vertebral fracture/subluxation. The neonate can present with hypotonia, quadriplegia or paraplegia; plain radiographs, ultrasound and MRI can aid diagnosis [19]. Hematomyelia, disruption of the spine, extraspinal hematoma and malalignment may be seen by the initial radiograph/bedside ultrasound and MR can further facilitate assessment of edema, ischemia or hemorrhage [20, 21].

Forceful hyperextension of the neck can occasionally result in ligamentous injuries at the craniocervical junction [6].

Carotid dissection has been described as a rare accompaniment of dystocic labor [22]; CT, Doppler ultrasound and MR may all have a role in diagnosis [22]. CT and MR of the brain may reveal findings of a stroke involving a carotid vascular territory; color Doppler of the carotid may reveal an intravascular flap suggesting dissection [6].

## Peripheral nerve injuries

**Fig. 13:**
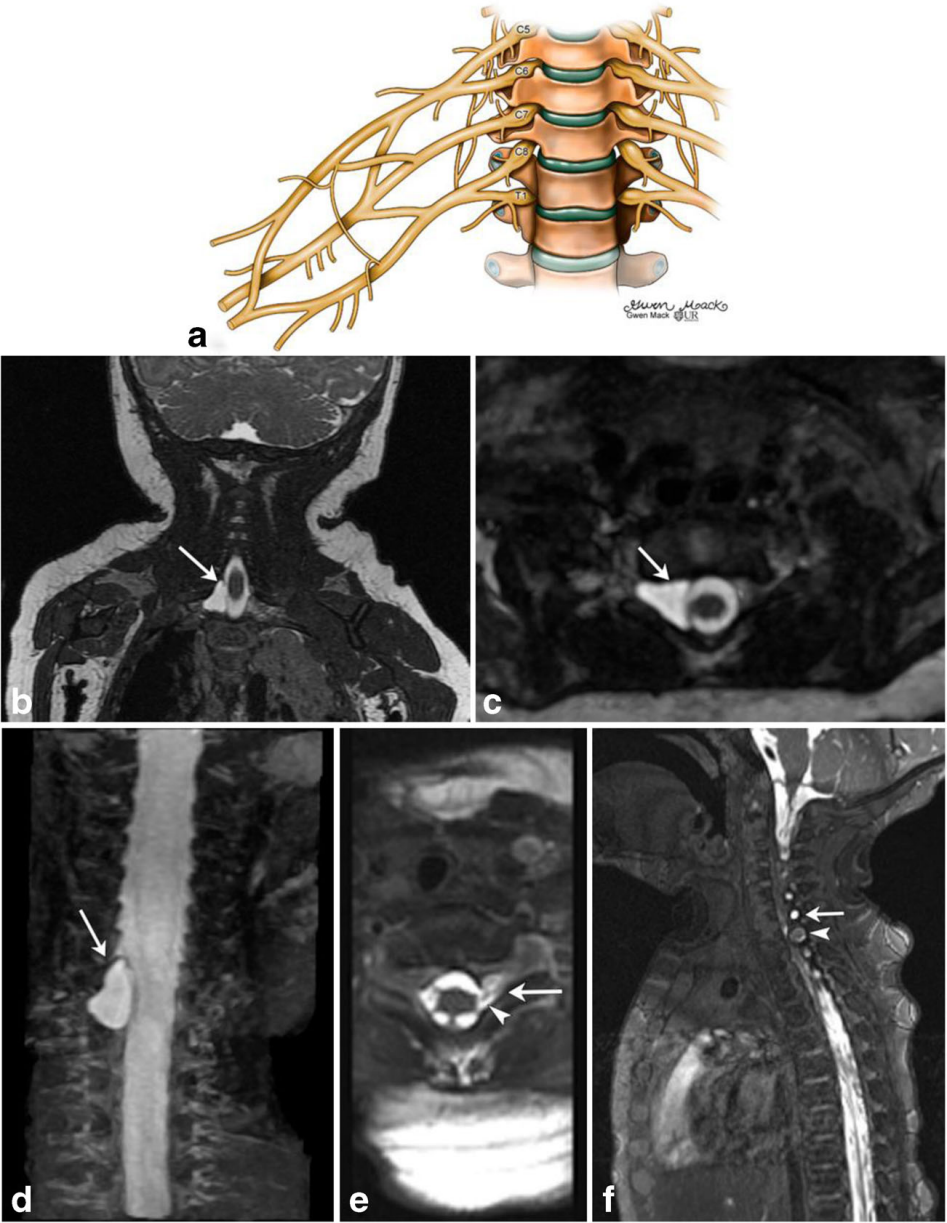
Peripheral nerve/Brachial plexus injury in two neonates Illustration (a) depicting normal brachial plexus. Image parts (b-f) High-resolution, steady state free precession (SSFP) MR images (b-d) in a 4-month-old boy with clinically suspected brachial plexus palsy shows a right-sided pseudomeningocele at C8-T1 level. The C8 nerve root was avulsed. Axial MR SSFP image of the cervical spine (e) in another 2-month-old female with left brachial plexus palsy demonstrated pseudomeningocele formation at the level of C8 (arrow) with disruption of the ventral nerve root (arrowhead). Sagittal MR SSFP image (f) shows disorganized soft tissue within the left C8 foramen with focal enlargement of the nerve at the exit of the neural foramen, consistent with neuroma formation (arrowhead). A smaller pseudomeningocele is also noted at C7 (arrow)

Birth-related neonatal brachial plexus injuries can occur prepartum or intrapartum. Incidence of obstetric brachial plexus palsy has been estimated at about 1 to 1.5 per 1000 live births in the United States [23]. The commonest fetal risk factor is macrosomia [23]; however, any maternofetal condition predisposing to fetal trauma including maternal obesity, maternal diabetes or instrumental delivery can be implicated [24]. Clavicular fractures often co-exist with brachial plexus injuries [23]. Cesarean can be protective [24], but does not exclude the likelihood of a brachial plexus injury [25]. The normal anatomy of the brachial plexus has been illustrated (Fig. 13a).

Involvement of C5/6 results in Erb’s palsy and lack of Moro’s reflex, whereas involvement of C7/T1 results in Klumpke’s palsy (Fig. 13b-f) and lack of Moro and grasp reflexes. Additionally, injury to T1 sympathetic fibers can lead to Horner’s syndrome. Complete plexus injury results in atonic limb and Horner’s sign [6].

This entity most commonly affects the upper trunk nerve components of the brachial plexus (C5-T1) [26]. This results in stretching, or less commonly avulsion of nerve roots. Avulsions, when they occur, usually localize to the C5 and C6 nerve roots and clinically manifest as Erb’s palsy [27]. High resolution heavily T2-weighted MR can show a traumatic pseudomeningocele (Fig. 13b-e), absent rootlets or roots (Fig. 13d-e) and abnormal spinal cord signal [6, 27].

Ultrasound has a role in preoperative evaluation of postganglionic brachial plexus in children with neonatal brachial plexus palsy; it demonstrated 68% sensitivity and 40% specificity for lower trunk involvement in a recent retrospective cohort study [28]. A small neuroma involving the upper trunk of the brachial plexus in an infant presenting with brachial plexus palsy was also recently described [29].

**Fig. 14:**
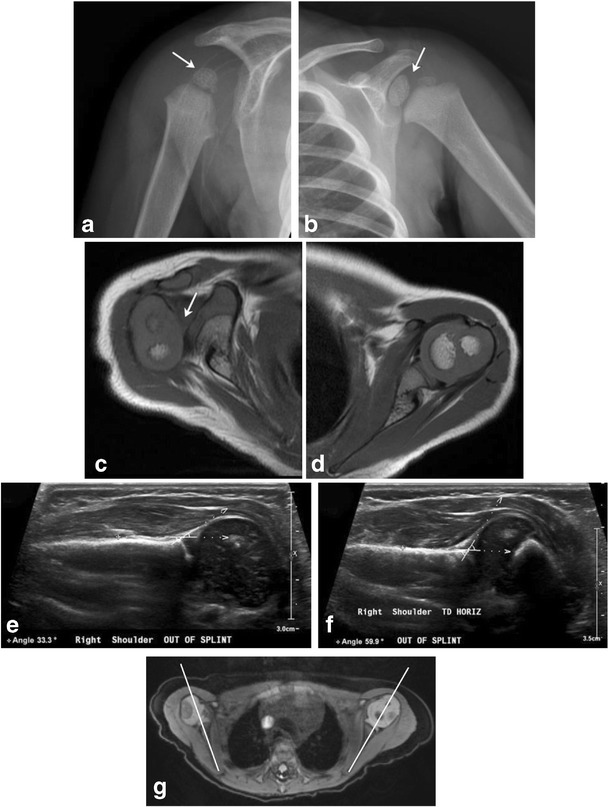
Glenohumeral dysplasia in two neonates Follow-up shoulder imaging in a 13-month-old child with clinically suspected brachial plexus palsy at birth (a-c). He subsequently developed glenohumeral dysplasia. The right humeral head is small, glenoid is shallow as seen on the radiograph (a); compare with the normally formed left humeral head and glenoid labrum (b). Axial T1 MR image (c) reveals posterior subluxation of humeral head; compare to normally aligned left humeral head (d). 2-month-old with history of right brachial plexus injury post-delivery (e-g). Ultrasound images from two exams performed 1 month apart (e and f) demonstrate progressive right glenohumeral dysplasia with interval increase in right humeral alpha angle from 33 degrees to 60 degrees. Axial DESS image from a follow-up MR exam (g) shows a shallow right glenoid with posterior humeral head subluxation. Normal left glenohumeral joint

Over the long term, progressive glenohumeral deformity may result. A Swedish population-based study found persistent anomalies in approximately 25% patients with neonatal brachial plexus palsy [30]. These abnormalities include glenoid retroversion, posterior subluxation of humeral head, dysplastic glenoid cavity and dysmorphic and hypoplastic humeral head (Fig. 14a-g) among others [26]. MRI is the gold standard for glenohumeral joint evaluation, although ultrasound may be used for screening or for evaluating joint reduction in real-time [31].

**Fig. 15:**
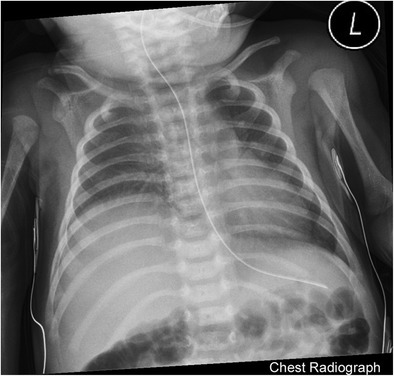
Neonatal Phrenic Nerve Palsy Chest radiograph of a 3-week-old with history of shoulder dystocia shows elevated right dome of diaphragm relative to left. Subsequently performed real-time ultrasound of the diaphragm (not included) revealed diminished excursion of the right hemidiaphragm relative to the left

Phrenic nerve palsy can occur as an accompaniment of traumatic brachial plexopathy. A retrospective review by Bowerson et al. [32] described the incidence of clinically significant phrenic nerve palsy in patients with brachial nerve palsy as 2.4%. Clinical manifestations may include respiratory compromise, lung infections, growth failure or even death [33]. Chest radiographs (Fig. 15) may reveal asymmetrical elevation of the affected diaphragm. Real-time chest ultrasound can accurately diagnose abnormal diaphragmatic motion on the affected side [34].

Radial nerve palsy can occur in context of a humeral shaft fracture [35].

Traumatic facial nerve injury can occur as a consequence of difficult extraction, particularly in context of forceps use. Prognosis for recovery is excellent; 90% recover completely [36].

Application of excessive traction to the head during breech delivery can result in unilateral recurrent laryngeal nerve injury and abductor paralysis. Left recurrent laryngeal nerve tends to be involved more often due to its longer course [37]. Prognosis for unilateral injuries is good, most usually resolve in 6 weeks, bilateral injuries tend to have a variable prognosis and some may require tracheostomy.

## Musculoskeletal injuries

Musculoskeletal injuries encompass both bony fractures and soft tissue injuries. Birth-related fractures in the newborn period, though overall rare, are important to recognize due to non-specific signs or symptoms, increased likelihood of missing them due to unossified cartilage and necessity of differentiating these from abusive trauma. These include fractures of both flat and long bones [38].

**Fig. 16:**
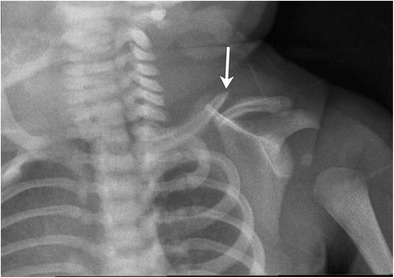
Clavicle fracture, shoulder dystocia Macrosomic infant of diabetic mother presented with mildly displaced fracture of the left clavicle after a delivery complicated by shoulder dystocia. Patient also had neonatal brachial plexus palsy from which he subsequently recovered

Clavicular fractures (Fig. 16) can occur with dystocic birth or with forceps delivery. Incidence is 2.7–5.7/1000 live births [6]. They can coexist with humeral fractures, traumatic brachial plexopathy and injuries to the phrenic and recurrent laryngeal nerve [6]. Besides the clavicle, fractures of other flat bones such as ribs, mandible and spine have also been described in the literature [6, 7].

Rib fractures have been described to be associated with dystocic birth. Based on a recent case series, birth-related rib fractures tend to be mid-posterior in location [39].

**Fig. 17:**
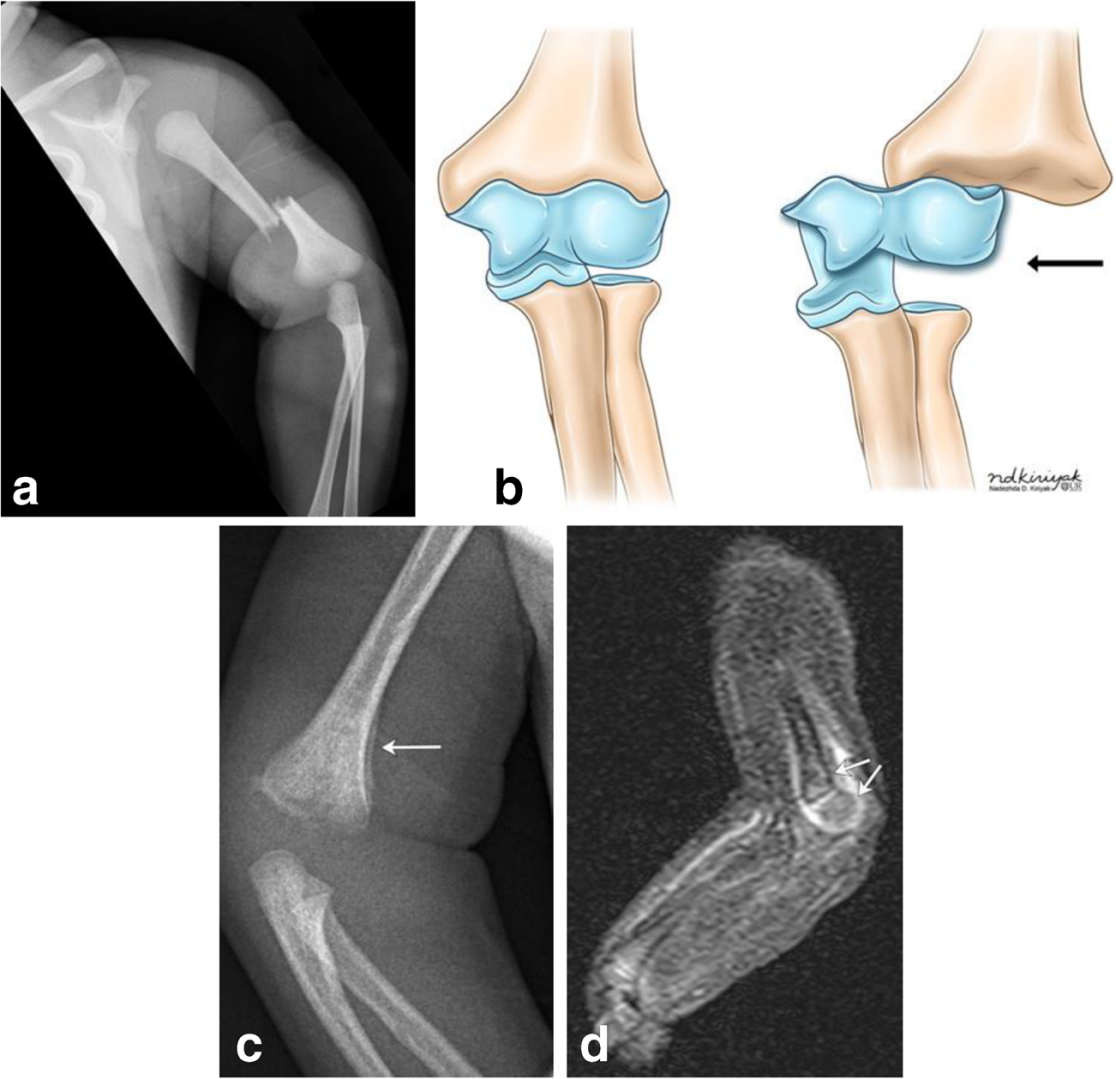
Humerus fractures as a consequence of traumatic birth Frontal radiograph of the left upper extremity in a 2-day-old infant demonstrates mid shaft fracture of the left humerus (a). Illustration (b) depicting chondro-epiphyseal separation at the distal humerus. Upon separation of the distal humeral epiphysis from the bone, it no longer lines up with the distal humerus (black arrow), as seen in figure parts c-d. 10-day-old male twin infant with history of traumatic delivery, presenting with decreased right arm movements. Right elbow radiograph (c) demonstrates fragmentation of the distal right humeral metaphysis, with mild periosteal new bone formation (arrow). Sagittal STIR MR image of the distal right humerus (d) demonstrates increased STIR signal and enhancement surrounding and involving the distal right humeral metaphysis and epiphysis, with mild posterior angulation of the distal epiphysis relative to the metaphysis (arrows), suggestive of distal humeral fracture with chondroepiphyseal separation

Humeral fractures can involve the humeral shaft (Fig. 17a) or the proximal or distal epiphyses [40]. Chondroepiphyseal separation of the distal humerus (classed at Salter I injury, Fig. 17b-d) can occur as a consequence of excessive traction on the upper extremity which may accompany a dystocic birth or one complicated by cephalopelvic disproportion [41]. The neonate can present with swelling/ pain and limitation of elbow movement, which is an important differentiating feature from the hypomobility of brachial plexus palsy. Also, a “muffled” crepitus can be present between the cartilaginous epiphysis and distal humerus [42]. Since unossified cartilage cannot be seen radiographically, these injuries are better appreciated by ultrasound. Alternatively, these injuries can be well seen by MR [42].

**Fig. 18:**
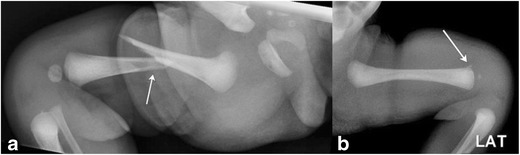
Femur fractures Frontal radiograph of the lower extremity in a 1-day-old infant (a) demonstrates displaced an oblique mid-shaft fracture of the right femur. This was a consequence of excessive traction on the femur. Lateral radiograph of the femur (b) in another 1-day-old breech infant demonstrates irregularity at the distal femoral metaphysis, which was proved to be a birth-trauma related physeal injury with chondroepiphyseal separation at the distal femur on the subsequently performed MR (images not included)

Femur fractures (Fig. 18a) though rare (incidence of 0.13 per 1000 live births) can occur in context of excessive traction on the femur; the most common manifestation being spiral fractures involving femoral shaft. Transphyseal fractures through the distal femur have also been described as a rare manifestation of birth trauma (Fig. 18b) [43]. Operative birth has been found to be associated with an increased incidence of these fractures, due to scarce available room for maneuvering with Cesarean births [44].

**Fig. 19:**
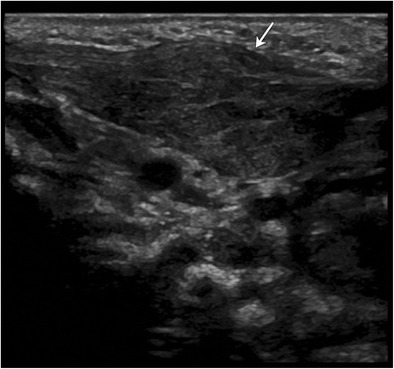
Fibromatosis colli Neck ultrasound obtained in a 6-week-old revealed fusiform enlargement of the left sternocleidomastoid without discernible underlying masses. Patient was diagnosed with fibromatosis colli. This patient had a history of dytocic birth

Sternocleidomastoid hematomas can be seen with a dystocic birth [45]. Alternatively, birth-trauma related venous ischemia of the sternocleidomastoid muscle has been postulated to result in benign fibroblastic proliferation of sternocleidomastoid muscle, also known as fibromatosis colli (Fig. 19) [46]. Usually unilateral with a right-sided predilection, this entity manifests between the first 4–8 weeks of life as a neck mass or torticollis [46]. Ultrasound (Fig. 19) is the imaging study of choice and demonstrates fusiform enlargement and heterogeneous echogenicity of the affected sternocleidomastoid muscle, which may appear “masslike” [46]. Biopsy is not universally recommended; most infants respond to physical therapy. Fine needle aspiration cytology (FNAC) is appropriate when the etiology of such a lesion is not clear or other diagnostic possibilities are also being considered. FNAC will show bland-appearing fibroblasts, degenerative, atrophic skeletal muscle, and muscle giant cells without inflammatory cells [47].

Operative intervention or botulinum toxin injection are considered in the rare circumstance where physical therapy fails [46].

## Visceral injuries

**Fig. 20:**
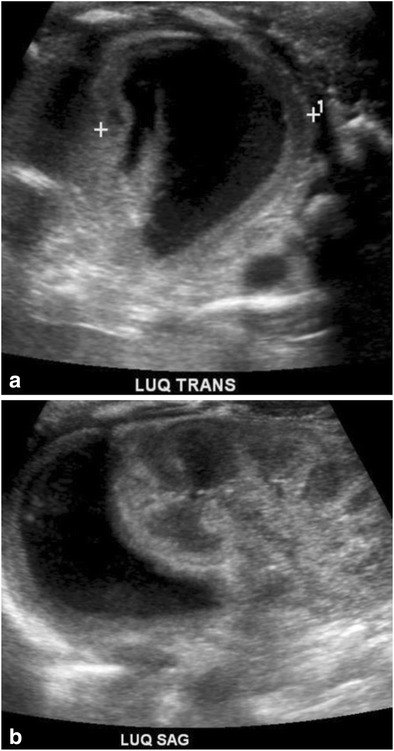
Visceral Injury Ultrasound of the left upper quadrant performed in a 3-day-old revealed a crescentic, near anechoic collection in the location of the left adrenal. Baby had a history of difficult birth characterized by prolonged 2nd stage of labor. Hematoma was followed by serial ultrasound and resolved at 13 weeks of age

Of the visceral organs affected by trauma, injuries to the liver [48, 49], spleen, kidney, adrenals and trachea have been described [50]. Neonatal adrenal hemorrhage (Fig. 20a, b) is rare and can be an important manifestation of birth-related mechanical trauma, found in only 0.2% of newborns.

Tracheal rupture can be anterior subglottic or distal tracheal in location. This rare and potentially fatal entity can occur in context of dystocic birth, and should be promptly suspected in neonates who develop subcutaneous emphysema or pneumomediastinum shortly after birth [51, 52]. Bronchoscopy should be expeditiously performed, and open surgical repair undertaken if necessary, especially in cases of distal tracheal rupture [51].

## Conclusion

Mechanical trauma related to birth can affect different organ systems of the neonate. While often of little clinical consequence, traumatic events can lead to cosmetic deformity, functional impairment and in extreme circumstances, even death. Imaging is important for detection, assignment of prognostic significance and follow-up, making it important for radiologists to be familiar with the imaging manifestations of these entities and their sequelae.

## Electronic supplementary material

ESM 1 (PDF 446 kb)

ESM 2 (PDF 64 kb)

ESM 3 (PDF 60 kb)

ESM 4 (PDF 71 kb)
