# Supplementary material for: Mechanical birth-related trauma to the neonate: An imaging perspective
Source: Insights Imaging. 2018 Jan 22;9(1):103–18. doi: 10.1007/s13244-017-0586-x (PMC5825313; doi:10.1007/s13244-017-0586-x)
Supplement: Supplementary file 1 — (PDF 446 kb) [file 13244_2017_586_MOESM1_ESM.pdf]

Classification of Birth-Related Trauma Based on Affected Organ System

Cranial

Extracranial

- **Scalp Injuries**
  - i. Caput succedaneum
  - ii. Subgaleal hemorrhage
  - iii. Cephalohematoma
- **Skull Fractures**
  - i. Linear
  - ii. Depressed
  - iii. Occipital osteodiastasis
  - iv. Leptomeningeal cyst
- **Facial Injuries**
  - i. Abrasions
  - ii. Facial fractures
  - iii. Retinal hemorrhages
  - iv. Facial nerve injuries

Intracranial

- **Extra-axial**
  - i. Epidural hemorrhage
  - ii. Subdural hemorrhage
  - iii. Subarachnoid hemorrhage
- **Intra-axial**
  - i. Parenchymal lobar hemorrhage / contusions
  - ii. Diffuse axonal / shear injury

Neck

- **Spinal Cord**
  - i. Hematomyelia
  - ii. Cord contusions
  - iii. Cranio-cervical junction avulsion
- **Vertebrae**
  - i. Fractures
  - ii. Dislocations
- **Carotid Dissection**

Peripheral Nerve Injuries

- **Brachial plexus** (including phrenic nerve palsy)
- **Radial nerve**

Musculoskeletal

- **Flat Bones**
  - – Clavicle
  - – Ribs
  - – Mandible
  - – Spine
  - – Facial bones
- **Long Bones**
  - – Humerus
  - – Femur
- **Sternocleidomastoid hematomas**
- **Skin abrasions**

Visceral Injury

- Liver
- Spleen
- Kidney
- Adrenal
- Trachea
