# Supplementary material for: Mechanical birth-related trauma to the neonate: An imaging perspective
Source: Insights Imaging. 2018 Jan 22;9(1):103–18. doi: 10.1007/s13244-017-0586-x (PMC5825313; doi:10.1007/s13244-017-0586-x)
Supplement: Supplementary file 2 — (PDF 64 kb) [file 13244_2017_586_MOESM2_ESM.pdf]

Birth-related Extracranial (Skull/Scalp) Injuries in the Newborn

Type of Extracranial Traumatic Lesion	Location	Clinical Presentation	Complications	Treatment Required
Caput Succedaneum	Subcutaneous, no periosteal involvement, crosses skull sutures	Boggy scalp Edema, “chignon” appearance of scalp	None, resolves spontaneously	None
Subgaleal Hemorrhage	Subaponeurotic	90% cases associated with vacuum extraction, fluctuant, firm scalp mass, can spread downward to the eye	Can lead to serious blood loss	Usually none required. Blood transfusion in cases of hemorrhagic shock; epicranial aspiration with compression dressing
Cephalohematoma	Subperiosteal hemorrhage, confined by skull sutures	Tense, firm, painful scalp mass; can enlarge over the first few days; can be mistaken for depressed skull fracture on palpation, may be associated with skull fractures	Usually resolve spontaneously in few weeks, infected cephalohematomas extremely rare	Usually none, rarely aspiration
