# Supplementary material for: Mechanical birth-related trauma to the neonate: An imaging perspective
Source: Insights Imaging. 2018 Jan 22;9(1):103–18. doi: 10.1007/s13244-017-0586-x (PMC5825313; doi:10.1007/s13244-017-0586-x)
Supplement: Supplementary file 3 — (PDF 60 kb) [file 13244_2017_586_MOESM3_ESM.pdf]

Birth-related Skull Fractures in the Neonate

Pattern of Skull Injury	Clinical & Imaging Features
Linear Fracture	• Commonly involve parietal bone.<br>• Asymptomatic and heal spontaneously.<br>• May be associated with cephalohematomas, epi-and subdural hematomas, dural tears and cerebral contusions.<br>• CT with 3D reformats diagnostic.<br>• No treatment necessary.
Depressed Fracture	• May present as "ping-pong" fractures due to softness of calvarium.<br>• May be associated with intra-and extra cranial hematomas.<br>• CT with 3D reformats diagnostic<br>• May spontaneously elevate; surgical management sometimes required.
Occipital Osteodiastasis	• Usually occurs with breech birth.<br>• Disrupted synchondrosis between squamous and occipital portions of occipital bone.<br>• Associated posterior fossa hemorrhages<br>• CT with 3D reformats/ MR for posterior fossa imaging
Leptomeningeal Cyst/ Growing Fracture	• Enlarging fracture due to leptomeningeal entrapment at fracture site<br>• Resorption of fracture margins secondary to CSF pulsations<br>• CT with 3 D reformats diagnostic
