# Supplementary material for: Mechanical birth-related trauma to the neonate: An imaging perspective
Source: Insights Imaging. 2018 Jan 22;9(1):103–18. doi: 10.1007/s13244-017-0586-x (PMC5825313; doi:10.1007/s13244-017-0586-x)
Supplement: Supplementary file 4 — (PDF 71 kb) [file 13244_2017_586_MOESM4_ESM.pdf]

Birth-related Traumatic Intracranial Injuries

Type of Traumatic Hemorrhage	Location	Clinical Presentation/Diagnosis	Accompanying injuries	Course/ Complications	Treatment
**Epidural Hemorrhage**	Between dura and periosteum	If large, can present with bulging fontanel or suture separation; history of difficult delivery with instrumentation, CT diagnostic	Linear skull fracture	Usually small; if large, can present with bulging fontanel or suture separation	Usually observation is sufficient, occasionally evacuation may be necessary
**Subdural Hemorrhage**	Into the subdural space, typically at parieto-occipital convexity, suboccipital or interhemispheric. May passively extend infratentorially	If small, irritability; moderate-sized may present with signs of raised intracranial pressure and episodic staring; if large, nuchal rigidity, dilated pupils, respiratory failure and cardiac arrest. CT diagnostic	Cerebral contusions	Usually uncomplicated; no compression of infratentorial structures	Usually observation is sufficient
**Subarachnoid**	Into the subarachnoid space	Seizures, hypotonia, irritability, somnolence, focal neurological symptoms	Intraparenchymal hemorrhage/ other extracranial hemorrhages	Usually uncomplicated, occasionally result in adhesions	None necessary
**Cerebellar Contusion**	Cerebellar parenchyma		Occipital osteodiastasis		Serial lumbar punctures; furosemide/ acetazolamide administration
**Cerebral Contusion**	Cerebral parenchyma	Seizures	Extracranial hemorrhages, skull fractures (occasionally occur in context of an existing AVM or coagulopathy)	Cysts and porencephalic cavities can form- spastic diplegia may result	No specific therapy
**Ischemic Stroke**	Internal carotid artery vascular territory	Horner’s syndrome, cranial nerve palsies, seizures, hemiparesis	Skull fractures	Varies according to distribution/ manifestation of injury	Varies according to distribution/ manifestation of injury
